# Bilingual Proficiency Effects on Word Recall and Recognition

**DOI:** 10.3390/bs15040437

**Published:** 2025-03-28

**Authors:** Yaqi Wang, Kai Yang, Simin Zhou, Hao Zhang, Tinghui Ma, Xiujuan Shi, Wen Ma

**Affiliations:** 1 School of Foreign Languages and Literature, Shandong University, Jinan 250100, China; wangyaqi@sdu.edu.cn (Y.W.); yangkai@sdu.edu.cn (K.Y.); 202320255@mail.sdu.edu.cn (S.Z.); hao.zhang0099@sdu.edu.cn (H.Z.); markma@sdu.edu.cn (T.M.); 2 Center for Clinical Neurolinguistics, Shandong University, Jinan 250100, China

**Keywords:** bilingual proficiency effects, word recognition, free recall, lexical processing

## Abstract

This study investigates the effects of bilingual proficiency on word recognition and recall across different memory tasks, with a focus on Chinese–English bilinguals. Participants learned lists of words in either their L1 (Chinese) or L2 (English) language while performing a semantic judgment task. Their memory for the learned words was subsequently assessed using three distinct tasks: a word recognition task (Experiment 1), a picture endorsement task (Experiment 2), and a free recall task (Experiment 3). The results revealed a significant L2 advantage in word recognition, as evidenced by higher hit rates, lower false alarm rates, and greater discrimination scores for L2 words. Furthermore, altering the retrieval cues from words to pictures led to a significant decrease in memory performance, but this did not diminish the L2 advantage. However, removing retrieval cues entirely eliminated the L2 advantage: participants demonstrated similar levels of correct recall for both L1 and L2 words, but showed a higher frequency of false recall for L1 words. To account for these dissociations between recall and recognition tasks, a level-based bilingual cognitive efficiency framework was proposed, incorporating factors such as pre-experimental exposure, cognitive resource allocation, the strength of lexical associations, and the demands of retrieval cues.

## 1. Introduction

With the increasing impact of globalization and the rise in bilingual education, the prevalence of bilingualism has grown significantly since the 1990s (Sánchez-Pérez & Manzano-Agugliaro, 2021; Liu et al., 2024). Bilingual individuals encode and retrieve daily experiences through both their dominant and non-dominant languages, which may influence their memory processes (Beato & Arndt, 2021; X. Ma et al., 2020; Messer et al., 2015). However, it remains unclear whether and how the effects of language dominance vary across different types of memory measures (Francis & Strobach, 2013; Francis & Gutiérrez, 2012; Vander Beken & Brysbaert, 2018). Therefore, in the present study, we investigate how recall and recognition memory differ for words presented in dominant (L1) and non-dominant languages (L2) among Chinese–English bilinguals.

In this study, we define L1 and L2 not strictly by their order of acquisition, but rather as the more and less proficient languages, or the dominant and non-dominant languages, respectively. It is important to note that this distinction may not always align with the chronological order of language acquisition. Additionally, the term “language proficiency effects” refers to within-participant differences in proficiency between L1 and L2.

### 1.1. Language Proficiency Effect in Recall and Recognition

The distinction between recall and recognition has long been a central topic in experimental psychology (Atkinson & Juola, 1974; Cox et al., 2018; Flexser & Tulving, 1978; Freund et al., 1969; Haist et al., 1992; Hollingworth, 1913; Jones, 1984; Lohnas & Kahana, 2013; Mandler, 1980; Rhodes et al., 2019). They are thought to rely on different cognitive mechanisms: recognition memory involves both undifferentiated familiarity strength and the recollection of qualitative details from the original study episode, whereas recall is believed to depend solely on recollection (Atkinson & Juola, 1974; Dobbins et al., 1998; Henson et al., 1999; Mandler, 1980; Toth, 1996; Yonelinas et al., 2005). Beyond their cognitive differences, the dissociation between recall and recognition performance has been widely documented (Francis et al., 2019; Boris & Parker, 2000; Hendry & Tehan, 2005; Serino et al., 2025; Srivastava, 2013; Wilma et al., 2019). A prominent example of this dissociation is the word frequency paradox: in free recall tasks, memory performance for high-frequency words is better than for low-frequency words (Criss et al., 2011; Badham et al., 2017; Balota & Neely, 1980; Glanzer & Bowles, 1976; Gregg, 1976; Malmberg & Nelson, 2003; Mandler et al., 1982), while the reverse is observed in recognition memory tasks (Francis & Gutiérrez, 2012; Francis & Strobach, 2013; Mizrahi et al., 2021).

Similar to the word frequency paradox, language proficiency can also lead to dissociations in recall and recognition performance (Francis & Gutiérrez, 2012; Francis & Strobach, 2013; Mizrahi et al., 2021; Vander Beken & Brysbaert, 2018). Specifically, L2 words tend to be better recognized, while L1 words are more effectively recalled. Several studies have documented an L2 advantage in word recognition (Francis & Gutiérrez, 2012; Francis & Strobach, 2013; Mizrahi et al., 2021). For instance, Francis and Strobach (2013) found that Spanish–English bilinguals showed higher hit rates and lower false alarm rates for L2 word recognition.

However, these studies assessed recognition memory using original words as retrieval cues. This method allows recognition to occur based solely on visual properties, without requiring semantic processing (Brainerd et al., 2022). As a result, participants may recognize an old L2 word even if they are unfamiliar with its meaning. The observed L2 recognition advantage might diminish if the direct visual match between encoded information and retrieval cues is eliminated. To test this hypothesis, we used object pictures as retrieval cues instead of words. Participants were asked to identify whether the objects depicted in the pictures had been mentioned during the learning phase. Furthermore, previous studies (Wang & Gennari, 2019) have shown that language effects on episodic memory occur only when verbal cues are presented during retrieval and diminish when nonverbal cues, such as pictures, are used. Therefore, the L2 advantage in recognition may disappear when pictures are used as retrieval cues.

In contrast, studies examining free recall have shown an L2 disadvantage, with poorer recall performance for words in the less frequently used language (Dhaene & Woumans, 2023; Francis & Baca, 2014; Francis et al., 2020; Vander Beken & Brysbaert, 2018). Nevertheless, Durgunoǧlu and Roediger (1987) found that word recall accuracy was independent of language. The inconsistent findings regarding the L2 disadvantage in word recall may be attributed to the selection of materials. Notably, previous studies did not control for participants’ knowledge of the meanings of L2 words. Difficulties in recalling or generating L2 words could stem from a lack of familiarity with their meanings (Brandt et al., 2005; Ning et al., 2018; Van Kesteren et al., 2014). Consequently, when participants are unfamiliar with the meanings of certain L2 words, their recall performance for these words is disadvantaged. However, this disadvantage may diminish when participants possess adequate knowledge of the L2 words’ meanings. Therefore, in the current study, we will control for L2 word knowledge during material selection and conduct a post-test analysis to assess participants’ knowledge with each L2 word in the experimental list.

### 1.2. Models Account for Language Proficiency Paradox

Since the 1970s, various computational models have been developed to account for the dissociation between recall and recognition memory (Anderson & Bower, 1972; Gillund & Shiffrin, 1984; Healy & Kubovy, 1978; Kimball et al., 2007; Kintsch, 1968; Reder et al., 2000; Norman & O’Reilly, 2003; Raaijmakers & Shiffrin, 1981; Sirotin et al., 2005). Among the most relevant to the current study are the Bind–Cue–Decide Model of Episodic Memory (BCDMEM; Dennis & Humphreys, 2001), the Source of Activation Confusion Theory (SAC; Diana & Reder, 2006), the Associative Memory Model (SAM; Raaijmakers & Shiffrin, 1981), and the Generate–Recognize Theory (GRT; Kintsch, 1968; Anderson & Bower, 1972).

The BCDMEM and SAC models have primarily been applied to explain L2 advantages in recognition tasks (Dennis & Humphreys, 2001; Reder et al., 2000). According to the BCDMEM model, L1 words are encountered more frequently in daily life than L2 words, leading to greater interference from pre-experimental contexts (Dennis & Humphreys, 2001; Myung et al., 2007). Similarly, the SAC model posits that the familiarity baseline for L1 words is higher than that for L2 words, making it more difficult for participants to retrieve the source of activation for L1 words. Consequently, this results in higher false alarm rates and lower hit rates for L1 words.

SAM and GRT are computational models that can be applied to explain the recall performance in language proficiency paradox. According to SAM, L1 words tend to form stronger associations with other items encountered either during pre-experimental exposure or within the experiment itself (Francis et al., 2019, 2020). As a result, L1 words are more effective than L2 words in cueing other items (Gillund & Shiffrin, 1984). This cueing advantage for L1 words leads to superior performance in recall tasks (Criss et al., 2011; Ratcliff et al., 1990; Shiffrin et al., 1990). Nevertheless, GRT proposes that high-frequency words or L1 words can be more easily recalled because greater exposure to these words facilitates their generation as retrieval candidates, compared to low-frequency or L2 words.

Models explaining the language proficiency paradox are largely adapted from models accounting for the word frequency paradox (Dennis & Humphreys, 2001; Reder et al., 2000). Since L1 words are used more frequently than their L2 equivalents, language proficiency is thought to influence lexical processing through mechanisms similar to those of word frequency (Gollan et al., 2008; Francis et al., 2020). However, the relationship between L1 and L2 words is not fully analogous to that between high- and low-frequency words. Specifically, high- and low-frequency words differ both in their conceptual representations and word forms, while dominant and non-dominant language words share conceptual representations but differ primarily in their word forms (Francis et al., 2019; Kroll & Stewart, 1994). Thus, a central aim of this research is to discuss the roles of word form and conceptual representations in the processes of word recognition and recall.

### 1.3. False Recall in L1 and L2

Memories are not exact replicas of original experiences (Lim & Goh, 2019; Wang et al., 2018). Instead, remembering is influenced not only by memory failures but also by inaccuracies and distortions (Abadie & Camos, 2019; Payne et al., 2009). Previous investigations into the language proficiency paradox, however, have predominantly focused on correct recall, overlooking the occurrence of false recall.

Several studies have investigated false recognition in L1 and L2 using the Deese–Roediger–McDermott (DRM) paradigm (Arndt & Beato, 2017; Beato & Arndt, 2021). The results showed that false recognition of the critical non-studied gist word was less frequent in L2 than in L1 (Suarez & Beato, 2023; Sıtkı et al., 2024). This was explained by a combination of the activation-monitoring framework (AMF) and the revised hierarchical model (RHM): because conceptual links in L1 are stronger than those in L2, L1 word activation spreads to related concepts more automatically through a well-organized and strongly associated network. In contrast, L2 word activation spreads to related nodes more slowly via weaker connections. As a result, it is more difficult for subjects to correctly retrieve the source of activation for L1 words than for L2 words, leading to more false memories in L1 than in L2 (Suarez & Beato, 2023).

The language proficiency effects observed in the DRM paradigm (Lim & Goh, 2019; Sahlin et al., 2005; Wang et al., 2018), however, may not be directly applicable to traditional word list learning experiments (Francis & Gutiérrez, 2012; Francis & Strobach, 2013). In the DRM paradigm, false memories are elicited by presenting a series of semantically related words, which leads to gist-based false recognition—resulting from the construction of associations between experimental items (Deese, 1959; Roediger & McDermott, 1995). In contrast, traditional word list learning tasks (Francis & Gutiérrez, 2012; Francis & Strobach, 2013) involve items that are not designed to be semantically related. Thus, false memories in these tasks are not the result of gist-based errors but are instead due to the spreading activation of experimental words to semantically or phonologically related words, or interference from pre-experimental experiences. Therefore, this study also aims to analyze and discuss how language proficiency influences false word recall.

### 1.4. Current Study

The primary aim of this study was to investigate how language proficiency affects word memory. Specifically, we first explored whether a dissociation exists between recall and recognition memory as a function of language proficiency, controlling for the confounding effect of L2 word knowledge. Furthermore, we examined whether L2 recognition advantages persist when there is no direct visual match between the retrieval cues and the learned items, requiring reliance on conceptual representations. Addressing this question will provide insight into the roles of word form and conceptual representations in shaping the effects of language proficiency on memory. Finally, we investigated whether false recall varies as a function of language proficiency.

To address these questions, we conducted three experiments to examine participants’ recall and recognition memory for L1 and L2 word lists. In all experiments, participants first learned 60 target words in either Chinese (L1, dominant language) or English (L2, non-dominant language). They then completed one of the following memory tasks: a word-cued recognition test (Experiment 1), a picture-cued endorsement task (Experiment 2), or a free recall task (Experiment 3). Finally, participants underwent a post-test to assess their knowledge of L2 word meanings.

Consistent with previous research, we hypothesized a dissociation in language proficiency effects across different memory measures. Specifically, we predicted that L2 words would be more accurately recognized in the word recognition task (Experiment 1). However, we anticipated that this effect may diminish when pictures were used as retrieval cues (Experiment 2) and could even reverse when no retrieval cues were provided (Experiment 3). Moreover, we expected memory performance to significantly decline when pictures, rather than words, were used as retrieval cues, as the influence of direct visual mapping was reduced. Finally, drawing on the Retrieval Hypothesis Model (RHM), we anticipated a higher rate of false recalls for L1 compared to L2.

## 2. Experiment 1

To ensure that the stimuli used in the current study would indeed produce an L2 advantage, as in previous research, we initiated the investigation by replicating Francis and Strobach (2013)’s study. In the experiment, participants learned words in either their L1 or L2 language, followed by the word recognition task. In line with previous research, we anticipated an L2 advantage in word recognition performance.

### 2.1. Method

#### 2.1.1. Participants

According to Kühberger et al. (2014), the typical effect size in psychological research is d = 0.4, and a well-powered study should aim for a power level of 80% (Cohen, 1962). A power calculation with this effect size therefore was performed via G*Power (Faul et al., 2009). The result indicated that a sample of 52 participants would be sufficient to observe the effect in a one-way ANOVA test (with *α* = 0.05 and *power* = 0.8). Additionally, several previously reported studies similar to the current research have found language effects in word recognition tasks, with approximately 30 participants per list (Francis & Baca, 2014; Francis & Strobach, 2013; Francis et al., 2018). We therefore aimed to recruit about 52–60 participants per list. In addition, the alpha of this study was set to 0.05 (two-tailed).

A total of 66 Chinese–English bilinguals were recruited from Shandong University of China. Five participants were excluded from the data analysis due to word recognition accuracy below two standard deviations from the group mean. Therefore, 29 participants were assigned to list 1 and 32 to list 2. All subjects were aged between aged between 17–22 (*Mage* = 18.62, *SDage* = 0.78, 11 males). In addition, they were all neurologically healthy and had either normal or corrected-to-normal vision. All experiments reported here were approved by the Ethics Committee of the School of Foreign Languages and Literature at Shandong University. Informed consent was obtained from all participants prior to their participation. They were awarded course credits or a small payment upon the completion of the study.

Participants provided self-ratings of language proficiency and exposure via the Language Experience and Proficiency Questionnaire (see Table 1) (LEAP-Q; Marian et al., 2007). All participants had Chinese as their first language and lived in a monolingual society in which Chinese is commonly used. In addition, they had acquired English as their second or third language.

#### 2.1.2. Material

The experimental stimuli consisted of 60 Chinese words and their English translation equivalents, evenly divided between living and nonliving categories. Each word set was paired with a corresponding black-and-white line drawing (572 × 572 pixels) representing the object described. Sixty filler items were also constructed, matched to the experimental stimuli in terms of word frequency and semantic category. All experimental words were nouns of concrete objects selected from the English Syllabus for the College Entrance Examination in China (D. G. Ma, 2022). The experimental words were monosemic, high-frequency items commonly encountered in everyday language exposure (M = 839.14, SD = 2048.88; Cai & Brysbaert, 2010). The letter length of the words ranged from 3 to 10 letters, with a mean length of 5.72 letters.

To optimize and validate the stimuli, an additional 20 participants, who had not participated in the main experiment, were recruited for a series of stimulus pre-tests. They were matched with the language background of the main sample participants. Difficulty in extracting English word meaning: Participants were required to rate how easily they could extract the meaning of the presented isolated English words using a 7-point scale (1 = extremely easy; 7 = extremely difficult). The results indicated that participants were generally able to extract word meanings with ease (*M* = 1.46, *SD* = 0.48). Difficulty in picture recognition: In this task, participants were presented with the experimental images alongside corresponding Chinese words describing the content. They were asked to rate the difficulty of recognizing the objects in the pictures on a 7-point scale (1 = extremely easy; 7 = extremely difficult). The results suggested that picture recognition was relatively easy (*M* = 1.36, *SD* = 0.53). Picture-word match rating: Participants were also asked to evaluate the extent to which the words matched the content of the pictures, using a 7-point scale (1 = do not match; 7 = extremely well-matched). The results indicated a strong match between the words and the images (*M* = 6.72; *SD* = 0.36). Object familiarity rating: Additionally, participants rated how frequently they encountered the objects depicted in the images in their daily lives, using a 7-point scale (1 = very rare; 7 = very often). The results showed that participants were generally familiar with the objects (*M* = 6.64, *SD* = 0.41).

#### 2.1.3. Design and Procedure

The study employed a one-way between-subjects design, with the language type of the learned words (L1 vs. L2) as the independent variable. Participants were randomly assigned to one of two groups: one group was tasked with learning and memorizing a list of L2 words, while the other group was assigned a list of L1 words.

Prior to the experiment, participants completed a consent form and an online language assessment using the Language Experience and Proficiency Questionnaire (LEAP-Q; Marian et al., 2007).

**Figure 1:**
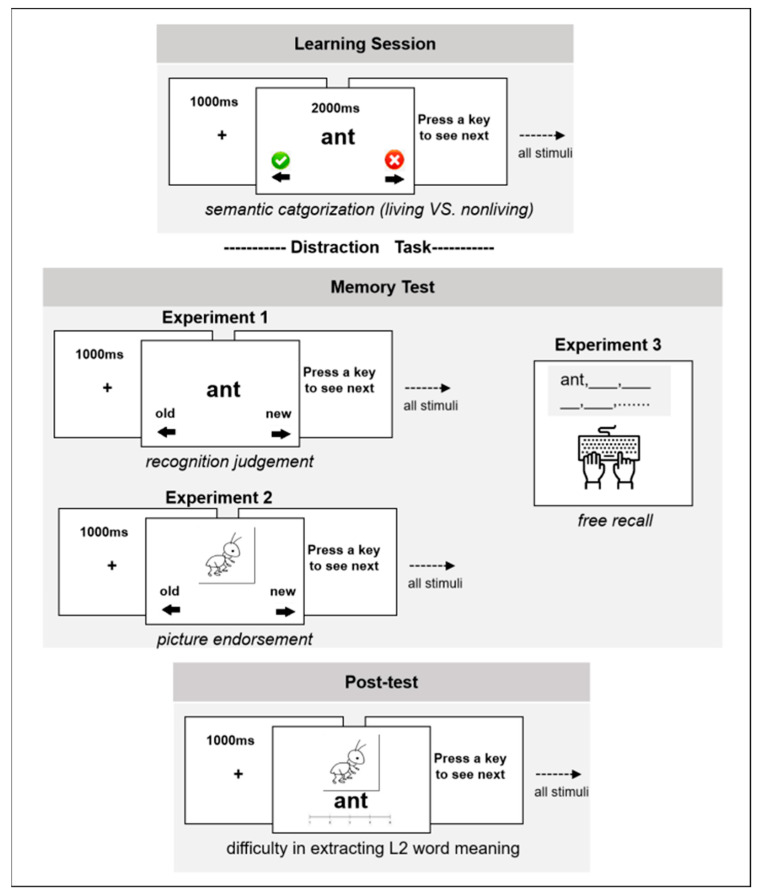
Schematic representations of the procedures in Experiments 1, 2, and 3.

During the learning session, participants received brief instructions before being presented with 60 randomly ordered words in either Chinese or English. Each trial began with a 1000 ms fixation cross, followed by the display of a target word for 2 s, during which participants were asked to memorize the word for a later memory test. Meanwhile, to ensure participants’ focus on the task and attention to word meaning, they were instructed to make semantic judgments (living vs. nonliving) about each word as quickly as possible (see Figure 1).

Following a 5 min distraction task involving the color verification of Chinese characters, participants completed a memory test of the learned words. In experiment 1, participants were asked to identify whether the presented words had been seen during the learning phase. Participants were informed that all previously presented words would appear in their original language. Each trial began with a 1 s fixation cross, followed by the presentation of a target word. Once a recognition judgment was made, the trial ended and the next one began. In total, participants were presented with 120 words in random order, half of which were previously learned and the other half new.

Finally, to evaluate each participant’s ability to recognize the individual English words used in the experiment, a post-test was conducted involving all the English words from the recognition task. Participants were asked to rate the difficulty of recognizing the meaning of each word on a 5-point scale, with 1 indicating ‘very easy’ and 5 indicating ‘very difficult’.

The experiment was conducted using PsychoPy 3.0 (Peirce et al., 2019) on computers with a 600 MHz processor. The monitor resolution was 1920 × 1080 pixels, with a refresh rate of 75 Hz. The computer screen was positioned at a viewing distance of approximately 60 cm.

#### 2.1.4. Data Treatment

According to signal detection theory, four primary dependent measures were computed to assess participants’ ability to discriminate between target and noise stimuli: hit rate, false alarm rate, and d-prime (d’) (Macmillan & Kaplan, 1985; Snodgrass & Corwin, 1988). The hit rate was defined as the proportion of correctly identified old items in the memory test, calculated by dividing the number of successful detections by the total number of old items. The false alarm rate was defined as the proportion of new items incorrectly identified as old, computed by dividing the number of incorrect identifications by the total number of new items in the memory test. Additionally, d’ served as a measure of an individual’s signal detection ability, computed as the difference between the z-scores of the hit rate and the false alarm rate. When either the hit rate or false alarm rate reached extreme values (i.e., 0 or 1), adjustments were made by replacing 0 with 0.5/n and 1 with (n − 0.5)/n, where n represents the total number of signal or noise trials (Macmillan & Kaplan, 1985).

To control for the influence of unfamiliar L2 words on the memory test, any English word trial was excluded if the word received a difficulty rating of 4 or 5 for meaning extraction. For the reaction time analysis, only trials with correct responses were included, and reaction times shorter than 0.1 s or exceeding 2.5 standard deviations from the group mean were excluded.

Reaction time and accuracy were analyzed using R (R Core Team, 2021). Generalized/linear mixed models (GLMMs/LMMs) were constructed with the lme4 package (Bates et al., 2015), including fixed effects for language (English vs. Chinese) and crossed random effects for participants and items. Random intercepts and slopes were included for both participants and items. If the full model failed to converge, its complexity was progressively reduced by first removing item correlations, followed by the removal of item slopes. If convergence was still not achieved, participant correlations and slopes were sequentially eliminated until a successful model fit was obtained. Additionally, the lmerTest package was employed to assess the significance of the fixed main effects and interactions (Kuznetsova et al., 2017), using the Satterthwaite approximation for degrees of freedom to compare the nested models. Furthermore, effects of interest were assessed by likelihood ratio tests comparing the full model with the effect of interest to a model without this effect.

### 2.2. Result

#### 2.2.1. Semantic Judgement Task

The results of mixed-effects modeling examining the influence of language on semantic judgment demonstrated main effects of language on both response accuracy (*χ*^2^ (1) = 10.28, *p* < 0.01) and reaction times (*χ*^2^ (1) = 36.20, *p* < 0.001) (see Table 2). Specifically, accuracy in semantic judgment was notably lower for L2 words (*M* = 0.84, *SD* = 0.36) in comparison to L1 words (*M* = 0.93, *SD* = 0.26). Additionally, participants exhibited significantly longer reaction times for L2 words (*M* = 1.10, *SD* = 0.04) relative to L1 words (*M* = 0.89, *SD* = 0.05).

#### 2.2.2. Word Recognition Task

- Response reaction times

Mixed-effects modeling assessed the effect of language on word recognition, with response reaction times as dependent variables. Additionally, reaction times were analyzed separately for experimental and foil items. However, no significant effect of language on reaction times was observed for either experimental or foil items (*ps* > 0.05, see Table 3).

- Signal detection theory’s measures

**Figure 2:**
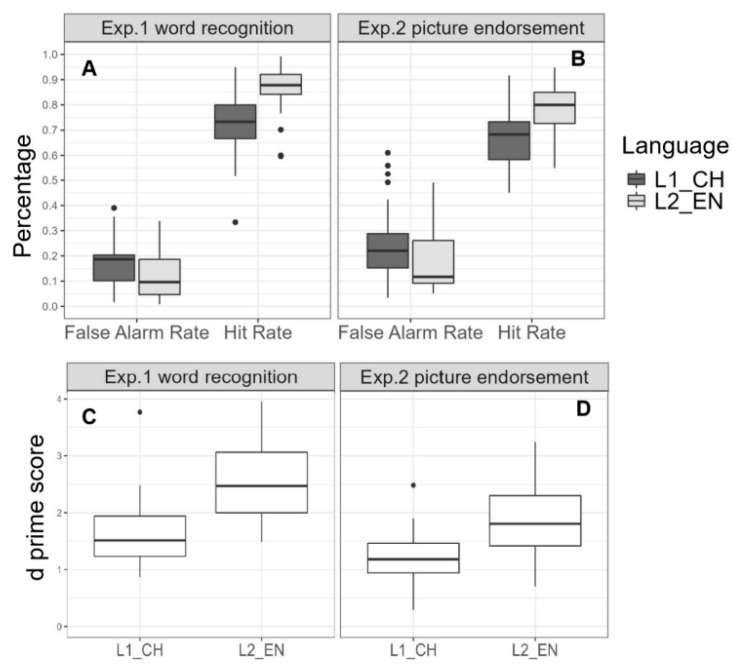
Memory performance as a function of language in Experiments 1 and 2. Panels (**A**,**B**) display the false alarm rates and hit rates as a function of language in Experiments 1 and 2, respectively. Panels (**C**,**D**) show d’ values as a function of language in the two experiments.

The word recognition task was also analyzed using one-way ANOVAs, with language (L1 vs. L2) as the independent variable and hit rate, false alarm rate, and d-prime for each participant as dependent variables. The results revealed the significant effect of language on hit rate (*F* (1, 59) = 28.41, *p* < 0.001, *η*^2^ = 0.33), false alarm (*F* (1, 59) = 6.71, *p* = 0.012, *η*^2^ = 0.10), and d-prime (*F* (1, 59) = 33.46 *p* < 0.001, *η*^2^ = 0.36) (see Figure 2). Planned pairwise comparisons revealed that participants achieved significantly higher hit rates in the L2 condition (*M* = 0.87, *SD* = 0.09) than in the L1 condition (*M* = 0.72, *SD* = 0.13), as well as higher d-prime values in the L2 condition (*M* = 2.58, *SD* = 0.69) compared to the L1 condition (*M* = 1.63, *SD* = 0.58) (see Table 4). In addition, false alarm rates were significantly lower in the L2 condition (*M* = 0.11, *SD* = 0.09) than in the L1 condition (M = 0.17, *SD* = 0.09).

### 2.3. Discussion

The results of Experiment 1 revealed that L2 words were recognized more accurately, despite their initial encoding and semantic judgment being generally less efficient than L1 words. These findings align with previous studies (Francis & Strobach, 2013; MacLeod & Kampe, 1996; Nott & Lambert, 1968; Reder et al., 2000), which demonstrated an advantage for L2 in word recognition, even after controlling for L2 word knowledge. This effect can potentially be explained by the SAC model, which will be discussed in detail in a later section.

Although L2 word recognition was associated with a higher hit rate, lower false alarm rate, and a higher discrimination score, it did not result in faster reaction times. One possible explanation for this discrepancy is that, although L2 words were better memorized (Dirix et al., 2020), their lexical processing was generally slower than that of L1 words (Cop et al., 2015; Dirix et al., 2020; Whitford & Titone, 2012), which may have offset the memory retrieval advantage.

Additionally, throughout the experiments, participants were exposed to both L1 and L2 words twice—once at encoding and again at retrieval. Consequently, participants may have based their recognition judgments primarily on the similarity of low-level visual features encountered at both stages, rather than engaging in conceptual-level processing. Thus, the L2 advantage may disappear if the direct visual match between encoding and retrieval is eliminated. Moreover, the exposure to both L1 and L2 words at the retrieval stage could have introduced cue-based retrieval, potentially amplifying the language proficiency effect. As reported by Wang and Gennari (2019), language effects on episodic memory are observed only when verbal cues are presented at retrieval, but are absent when nonverbal cues, such as pictures, serve as retrieval cues. Therefore, it is plausible that the L2 advantage diminishes when pictures, rather than words, are used as retrieval cues. To test this hypothesis, we conducted Experiment 2, which provides insight into the roles of word form processing and conceptual representations in memory recognition.

## 3. Experiment 2

In Experiment 1, we replicated the study by Francis and Strobach (2013) and found significant advantages for L2 in a word recognition task. However, as previously discussed, these results may have been influenced by the global visual similarity between word forms at the encoding and retrieval stages. To address this potential confounding factor, we employed pictures rather than words as retrieval cues in the current experiment. Based on the findings of Wang and Gennari (2019), we anticipated that the L2 advantage would disappear and that overall memory performance would decline for both L1 and L2.

### 3.1. Method

#### 3.1.1. Participants

Sixty-three additional subjects, who had not participated in Experiment 1, were recruited. Their language backgrounds were matched with those of the participants in Experiment 1 (*ps* > 0.1) (see Table 1). Four participants were excluded from the data analysis due to memory performance that was more than two standard deviations below the group mean. Consequently, the final sample consisted of 30 participants for the L1 list and 29 for the L2 list. They were aged between 17 and 21 (*Mage* = 18.59, *SDage* = 0.77; 10 males). Informed consent was obtained from all participants, and they received either a small payment or course credits.

#### 3.1.2. Material, Design, Procedure, and Data Treatment

The same stimuli, design, procedures, and data treatment used in Experiment 1 were adopted for this study with one alteration: in the memory test, black-on-white line drawings rather than words were used as the retrieval cues. Participants were instructed to identify whether the objects presented had been studied during the learning phase. In total, participants were exposed to 120 pictures during the memory test, half of which referred to a learned object, and the other half were new (see Figure 1).

### 3.2. Result

#### 3.2.1. Semantic Judgment Task

The mixed-effects modeling assessment of the effect of language on semantic judgment revealed significant main effects of language on both response accuracy (*χ*^2^ (1) = 9.96, *p* < 0.02) and reaction times (*χ*^2^ (1) = 46.44, *p* < 0.001). Accuracy in semantic judgment was significantly lower for L2 words (*M* = 0.88, *SD* = 0.12) compared to L1 words (*M* = 0.96, *SD* = 0.11) and reaction times were significantly longer for L2 words (*M* = 1.14, *SD* = 0.03) than for L1 words (*M* = 0.85, *SD* = 0.07) (see Table 2).

#### 3.2.2. Picture Endorsement Task

- Response reaction times

Consistent with the approach in Experiment 1, reaction times were examined independently for experimental and foil items. However, no statistically significant effect of language on reaction times was found for either experimental or foil items (*ps* > 0.05) (see Table 3).

- Signal detection theory measurements

Signal detection theory metrics were analyzed for the picture endorsement task using one-way ANOVAs, with language (L1 vs. L2) as the most independent variable and hit rate, false alarm rate, and d-prime as the dependent variables. Similarly to Experiment 1, the analysis demonstrated a significant main effect of language on both the hit rate (*F* (1, 59) = 19.21, *p <* 0.001, *η*^2^ = 0.25) and d-prime (*F* (1, 59) = 17.48, *p* < 0.001, *η*^2^ = 0.23). In contrast, the effect of on false alarm rates was marginally significant (*F* (1, 59) = 2.99, *p* = 0.089, *η*^2^ = 0.05). Planned pairwise comparisons indicated that participants exhibited significantly higher hit rates in the L2 condition (*M* = 0.79, *SD* = 0.11) compared to the L1 condition (*M* = 0.66, *SD* = 0.12), as well as higher d-prime values in the L2 condition (*M* = 1.87, *SD* = 0.70) compared to the L1 condition (*M* = 1.20, *SD* = 0.50). In addition, the participants showed a lower false alarm in the L2 condition (*M* = 0.18, *SD* = 0.13), contrasted with the L1 condition (*M* = 0.25, SD = 0.15), but the effect was marginally significant (*p >* 0.05) (see Table 4 and Figure 2).

#### 3.2.3. Comparisons Across Experiments 1 and 2: Cue Effect

- Response reaction times

The model analyzing reaction times for both experimental and foil trials in the memory test revealed the significant main effect of the retrieval cue (experimental trials: *χ*^2^ (1) = 47.14, *p <* 0.001; foil trials: *χ*^2^ (1) = 60.43, *p* < 0.001). For experimental trials, reaction times were significantly longer when pictures were used as retrieval cues (*M* = 1.20, *SD* = 0.49) compared to word cues (*M* = 0.94, *SD* = 0.36). Similarly, for foil trials, participants exhibited longer reaction times with picture cues (*M* = 1.46, *SD* = 0.58) than with word cues (*M* = 1.04, *SD* = 0.41). Nevertheless, the main effect of language and the interaction between language and retrieval cue did not reach statistical significance (*ps >* 0.05).

- Signal detection theory’s measures

Hit rate, false alarms, and d’ in memory tests were analyzed using a 2 (language: EV vs. CH) × 2 (retrieval cue: word vs. picture) between-subject ANOVA. For hit rate, significant main effects were found for both language (*F* (1, 116) = 47.16, *η*^2^ = 0.29, *p <* 0.001) and retrieval cue (*F* (1, 116) = 11.07, *η*^2^ = 0.09, *p* < 0.01). Hit rates were higher in the word condition (*M* = 0.80, *SD* = 0.14) than in the picture condition (*M* = 0.73, *SD* = 0.13). Additionally, hit rates for L2 words (*M* = 0.83, *SD* = 0.11) were higher than those for L1 words (*M* = 0.69, *SD* = 0.13). For false alarm rates, the word condition (*M* = 0.14, *SD* = 0.09) yielded lower rates compared to the picture condition (*M* = 0.21, *SD* = 0.14), *F* (1, 116) = 11.96, *η*^2^ = 0.09, *p* < 0.001. Furthermore, false alarm rates were lower under the L2 condition (*M* = 0.15, *SD* = 0.11) than under the L1 condition (*M* = 0.21, *SD* = 0.13), *F* (1, 116) = 8.13, *η*^2^ = 0.07, *p* < 0.01. Regarding d’, values were higher in the word condition (*M* = 2.13, *SD* = 0.80) compared to the picture condition (*M* = 1.54, *SD* = 0.69), *F* (1, 116) = 26.28, *η*^2^ = 0.19, *p* < 0.001. Additionally, d’ values were higher under the L2 condition (M = 2.24, SD = 0.78) than under the L1 condition (*M* = 1.42, *SD* = 0.58), *F* (1, 116) = 50.17, *η*^2^ = 0.30, *p* < 0.001.

### 3.3. Discussion

In Experiment 2, retrieval cues were shifted from words to pictures, eliminating the direct visual match between encoding and retrieval. This manipulation resulted in a general decline in memory performance, as evidenced by a lower hit rate, a higher false alarm rate, a decreased d’ score, and slower reaction times. These findings highlight the essential role of direct visual matching of word forms in recognition, consistent with the encoding specificity principle (Tulving & Thomson, 1973). We will further elaborate on these effects in a later section.

The most critical finding of Experiment 2 is the partial replication of the L2 advantages observed in Experiment 1. Specifically, we found a significantly higher hit rate and discrimination score for L2 words. However, the false alarm rate was only marginally lower for L2 words compared to L1 words. These results suggest that L2 words are better remembered even in the absence of a direct visual match between encoding and retrieval, which contradicts our initial predictions. Nonetheless, this does not imply that the visual matching of word forms was entirely absent during retrieval. Rather, we propose that visual matching may occur indirectly, whereby corresponding words are extracted based on picture content and subsequently matched with encoded words. This process would then contribute to picture endorsement decisions. We will explore this point further in a later section.

In both Experiment 1 and Experiment 2, we observed L2 advantages in memory recollection when retrieval cues allowed for a direct or indirect visual match of word forms. However, it remains unclear how these L2 advantages are affected when visual matching between encoding and retrieval is entirely prevented. Previous studies have suggested an L1 advantage in word recall (Durgunoǧlu & Roediger, 1987; Glanzer & Duarte, 1971; Francis & Baca, 2014; Vander Beken & Brysbaert, 2018; Yoo & Kaushanskaya, 2016), although the evidence remains mixed (Francis et al., 2018; Vander Beken & Brysbaert, 2018; Yoo & Kaushanskaya, 2016). To further investigate this, Experiment 3 will examine memory performance for L1 and L2 words using a free recall task, in which no retrieval cues are provided, and recollection must rely solely on memory retrieval.

## 4. Experiment 3

In Experiments 1 and 2, we observed significant L2 advantages in both word recognition and picture endorsement tasks. However, it remains unclear whether and how language proficiency influences word recall (Francis & Baca, 2014; Francis et al., 2020; Han & Kim, 2017). To address this, in Experiment 3, we employed a free recall task in which no retrieval cues were provided. Additionally, to gain a comprehensive understanding of free recall, we will analyze and discuss both correct recall and false memory recollection. Based on previous research, we predicted that the L2 advantages observed in word recognition would shift to L1 advantages in recall, though L1 may also elicit more false recollections than L2.

### 4.1. Method

#### 4.1.1. Participants

Sixty-seven additional participants, who had not taken part in Experiments 1 and 2, were recruited. Their language backgrounds were matched with those of the participants from Experiments 1 and 2 (*ps* > 0.05; see Table 1). Two participants were excluded from the analysis due to a memory performance of more than two standard deviations below the group mean. As a result, the final sample comprised 33 participants in the L1 group and 32 in the L2 group. All subjects are aged between 17 and 22 (*Mage* = 18.27, *SDage* = 0.82; 13 males). Informed consent was obtained from all participants, who were compensated with either a small payment or course credit.

#### 4.1.2. Material, Design, and Procedure

The same stimuli, design, and procedures from Experiments 1 and 2 were employed in this study, with one exception: in the memory test, no retrieval cues were provided, and participants were instructed to recall and report all the words they could remember in their original language, typing their responses on a keyboard (see Figure 1).

#### 4.1.3. Data Treatment

First, all misspellings were corrected. Subsequently, all recollected words were categorized by the experimenter as either correct or false recollections. If a recalled word corresponded to an old word or a synonym of an old word, whether in its plural or singular form, it was considered a correct recollection. However, if a response referred to a new object, it was categorized as a false recollection.

The false recollection rate for each participant was calculated by dividing the number of false recollections by the total number of recollections. In addition, the overall correct recollection ratio was determined by dividing the number of correctly recalled words by the total number of learned words.

For data screening, participants who failed to recall any target words were excluded. Additionally, trials of L2 list were removed from all analyses if the corresponding L2 word was rated 4 or 5 on the difficulty of extracting its meaning.

### 4.2. Result

#### 4.2.1. Semantic Judgement Task

Mixed-effects modeling assessing the effect of language on semantic judgment revealed significant main effects of language on both response accuracy (*χ*^2^ (1) = 5.33, *p* = 0.02) and reaction times (*χ*^2^ (1) = 53.56, *p* < 0.001). Accuracy in semantic judgment was significantly lower for L2 words (*M* = 0.89, *SD* = 0.31) compared to L1 words (*M* = 0.93, *SD* = 0.26), and reaction times were significantly longer for L2 words (*M* = 1.11, *SD* = 0.28) than for L1 words (*M* = 0.86, *SD* = 0.25) (see Table 2).

#### 4.2.2. Free Recall Task

**Figure 3:**
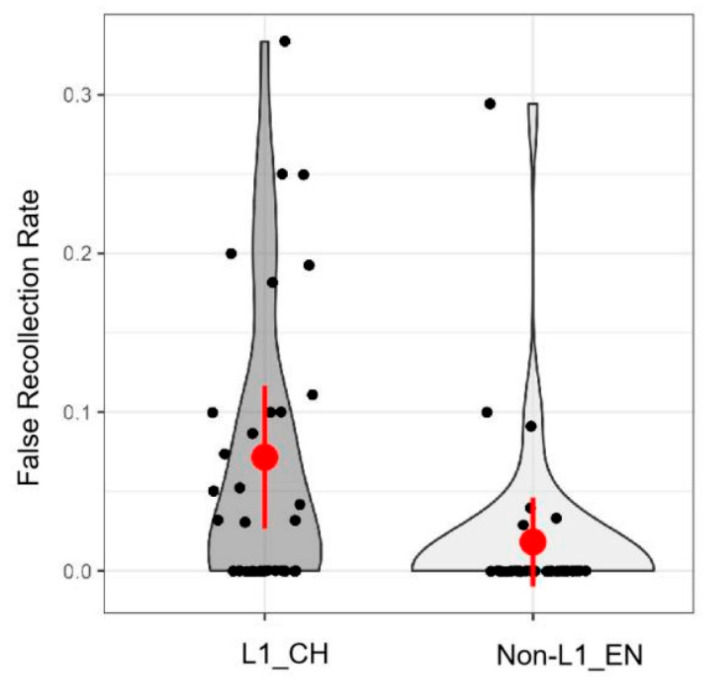
False recollection rate as a function of language. The false recollection rate was calculated by dividing the number of false recollections by the total number of recollections.

False recollection in the free recall task was analyzed using one-way ANOVAs, with language (L1 vs. L2) as the independent variable and the number of falsely recollected words and the false recollection rate as the dependent variables. The results revealed a significant language effect on both the number of falsely recollected words (*F* (1, 63) = 5.26, *p* < 0.05, *η*^2^ = 0.08) and the false memory rate (*F* (1, 63) = 7.53, *p* < 0.01, *η*^2^ = 0.11). Planned pairwise comparisons showed that participants exhibited more false memories under the L1 condition (number of false recollections: *M* = 0.88, *SD* = 1.05; rate of false recollection: *M* = 0.07, *SD* = 0.09) than under the L2 condition (number of false recollections: *M = 0*.3, *SD* = 0.93; rate of false recollection: *M* = 0.02, *SD* = 0.06) (see Figure 3). However, mixed-effects modeling on recall accuracy showed no significant effect of language. Additionally, there was no significant language effect on the total number of recalled words and correct recollection ratio (*ps >* 0.05) (see Figure 3).

### 4.3. Discussion

The results of the free recall task revealed no significant differences in correct recall between L1 and L2. These findings suggest that language proficiency effects on word memory diminish when retrieval cues are eliminated and no visual match between the encoding word form and retrieval cues can occur. However, the absence of language proficiency effects is inconsistent with previous research (Criss et al., 2011; Badham et al., 2017; Balota & Neely, 1980; Dhaene & Woumans, 2023; Gregg, 1976; Vander Beken & Brysbaert, 2018), which may be attributed to the control of compounding effects related to L2 word knowledge. We will explore this further in a later section.

Additionally, we found that participants recalled more unseen L1 words than unseen L2 words. This finding aligns with prior studies investigating bilingual false memory (Anastasi et al., 2005; Arndt & Beato, 2017; Beato & Arndt, 2021). The findings can be attributed to more efficient spreading activation and stronger conceptual associations for L1 words (Howe et al., 2009). We will discuss these results in greater detail in the following section.

## 5. Discussion

The present study investigated the influence of language proficiency on various memory measurements. We conducted three experiments testing memory for words in the dominant language (L1) and non-dominant language (L2) using word recognition, picture endorsement, and free recall tasks. The results showed that L2 words were better recognized, with a higher hit rate, lower false alarms, and higher d’ scores. However, using pictures as cues significantly decreased memory performance but did not alter the L2 advantage. Moreover, correct recall did not differ between L1 and L2 words, although false recall was more frequent for L1 words. These findings generally align with our prediction of a dissociation between recall and recognition, except for the observed language proficiency effects in the picture endorsement task and the absence of L1 advantages in recall accuracy. The following sections discuss the explanations and implications of these findings.

### 5.1. L2 Advantages on Word Recognition

We discovered a mirror effect in word recognition between L1 and L2 words, showing that words in the less proficient language are associated with fewer false recognitions and more correct recognitions in Experiment 1. These findings align with previous research (Francis & Gutiérrez, 2012; Francis & Strobach, 2013; Mizrahi et al., 2021) and can be explained by SAC theory (Buchler & Reder, 2007; Diana & Reder, 2006) and familiarity increment account (Dobbins et al., 1998; MacLeod & Kampe, 1996).

According to SAC theory, L1 words may have a higher fan effect than L2 words, as L2 words are linked to fewer pre-experimental contexts (Buchler & Reder, 2007; Diana & Reder, 2006; Francis & Strobach, 2013). As a result, during memory retrieval, there is less competition between prior and current contextual associations for L2 words, facilitating the retrieval of their encoding context and leading to more correct recognition instances. Additionally, the baseline familiarity level for L1 words is higher due to more pre-experimental exposure, making L1 foils more likely to surpass the familiarity threshold, resulting in more false recognition instances.

The L2 advantage in word recognition may also be attributed to differing increases in familiarity between L1 and L2 words. Specifically, incidental exposure to experimental words increases their familiarity, which in turn facilitates successful recognition (Hintzman, 1976). Furthermore, previous research suggests that low-frequency words receive a greater familiarity boost during study compared to high-frequency words, as they are encountered less often in everyday life, leading to a low-frequency advantage in recognition memory (Dobbins et al., 1998; MacLeod & Kampe, 1996). Similarly, L2 words may experience a greater increase in familiarity, contributing to the observed L2 advantage.

Both the SAC model and the familiarity increment account stem from theories addressing the word frequency paradox, with a common focus on the role of pre-experimental experiences. While these models offer plausible explanations for L2 advantages in word recognition, they do not clearly differentiate between recognition based on word form and recognition based on conceptual representations. For instance, it remains uncertain whether the recognition advantage of a low-frequency word like “otter” over a high-frequency word like “mouse” arises from the rarity of encountering the actual animal or from reduced exposure to the word form or spelling prior to the experiment. Comparing L1 and L2 word memory provides a means to disentangle word-level from conceptual-level processing, as both languages share the same conceptual representations but differ in word forms (Brysbaert & Duyck, 2010; T. Dijkstra & van Heuven, 2002). Therefore, the observed recognition advantage in less frequent or less proficient languages should theoretically be attributed primarily to word-level processing. We will further discuss this in the following section.

### 5.2. Bilingual Memory in Picture Endorsement Task

Experiment 1 demonstrated a significant L2 advantage in word recognition, while Experiment 2, which used pictures as retrieval cues, showed a general decline in memory performance but maintained the L2 advantage. These findings highlight the critical role of retrieval cues in memory recall.

The persistence of the L2 advantage with picture cues raises an important question: how does this advantage manifest when word forms are absent? One plausible explanation is that participants indirectly process word forms by extracting conceptual information from pictures, generating corresponding words, and then making familiarity-based decisions. As discussed, L2 words may benefit from greater familiarity boosts and reduced interference, enhancing their recognition. Alternatively, the L2 advantage might result from superior conceptual representations of L2 words. During retrieval, participants extract conceptual information from the picture cues and make endorsement judgments based on their familiarity with these conceptual representations. However, prior research suggests that the connection between L2 words and their semantic concepts is weaker compared to L1 (Kroll & Stewart, 1994), and lexical processing is generally less efficient for L2 than for L1. Therefore, it is unlikely that the L2 advantage arises from conceptual-level processing.

Using pictures as cues also eliminated the direct visual match between encoded and retrieved word forms, reducing memory performance, consistent with the encoding specificity principle (Grant et al., 1998; Tulving & Thomson, 1973). In Experiment 2, participants first had to recognize the conceptual meaning of pictures before generating words. Failure to generate the correct words or recognize the objects would prevent accurate judgments. In contrast, Experiment 1 allowed for direct word form matching, reducing the cognitive load. Moreover, picture cues might prompt participants to generate multiple words (e.g., “bird”, “parrot”, “cockatoo”), increasing interference and processing cost.

Lastly, while the false alarm rate was significantly higher in the word recognition task, it only approached marginal significance in the picture endorsement task. This difference may be attributed to variations in cue-elicited spreading activation. In Experiment 2, since identical picture cues were used for both L1 and L2, the activated concepts were also identical. For instance, when participants viewed a picture of a “lion”, they might also think of related animals such as “tiger” or “cat”, potentially leading to a false sense of familiarity when encountering foils like “tiger” or “cat”. In contrast, in Experiment 1, where L1 or L2 words served as retrieval cues, the spreading activation during retrieval was language-dependent. Previous research suggests that spreading activation is more efficient and stronger in L1 (Arndt & Beato, 2017; Beato & Arndt, 2021; Howe, 2006), resulting in greater cue-elicited interference during L1 retrieval.

Overall, the results of Experiment 2 highlight the critical role of retrieval cues in bilingual memory. The conceptual information conveyed by the cues, their visual similarity to the encoded material, and the spreading activation they elicit all significantly influence memory retrieval.

### 5.3. Similar Recall Accuracy and More False Recall in L1 vs. L2

In the free recall task, no significant effects of language proficiency on recall accuracy were observed, contrary to our predictions. However, the analysis of false recalls showed that participants generated more novel L1 words than L2 words, aligning with our expectations.

The findings from Experiment 3 revealed that the false recall of L1 words occurred more frequently than the false recall of L2 words, consistent with previous research on bilingual false memory (Howe et al., 2009; Suarez & Beato, 2023; Gurrola & Francis, 2024). This pattern can be explained by RHM (Kroll & Stewart, 1994), which suggests that conceptual associations are generally stronger in L1 than in L2. Consequently, the encoding and retrieval of L1 words are more likely to activate related concepts via a well-organized and highly interconnected network. In contrast, L2 word activation spreads more slowly due to weaker conceptual links. This disparity in activation strength increases the difficulty of distinguishing the source of activation for L1 words, leading to a higher rate of false recall (Suarez & Beato, 2023). An alternative explanation is that participants may have produced more false recalls of L1 words simply because L1 words were more likely to be generated as retrieval candidates due to greater pre-experimental exposure, as proposed by GRT. If this were the case, we would expect a higher overall recall of L1 words compared to L2 words. However, this trend was not observed, suggesting that the increased false recall of L1 words cannot be fully explained by GRT.

In the free recall task, no significant effects of language proficiency on recall accuracy were observed when no retrieval cues were presented. This absence of language proficiency effects may be attributed to the nature of retrieval demands in free recall, which is considered a “conceptually driven” task that relies on stored conceptual representations (Jacoby, 1983; Durgunoǧlu & Roediger, 1987). During encoding, participants processed the conceptual-level information of both L1 and L2 words to complete the semantic judgment task. Since L1 and L2 words often share the same conceptual representation (Brysbaert & Duyck, 2010), the activation of this shared conceptual node during recall may have minimized any potential influence of language proficiency on recall accuracy.

The absence of language proficiency effects on correct recall contradicts previous findings (Francis & Baca, 2014; Francis et al., 2020; Dhaene & Woumans, 2023; Vander Beken & Brysbaert, 2018) but is consistent with the results of Durgunoǧlu and Roediger (1987). One possible explanation for these inconsistent findings is the failure to control for L2 word knowledge in earlier studies, as difficulties in recalling L2 words may arise from a lack of familiarity with the words themselves. In the present experiment, we addressed this issue by selecting highly familiar L2 words and excluding trials in which participants lacked knowledge of the L2 words. This approach minimized the difficulty of generating L2 words, thereby reducing the L2 disadvantages reported in previous studies. Our findings underscore the importance of controlling for L2 word knowledge in bilingual memory research.

### 5.4. Level-Based Bilingual Cognitive Resource Account

In the previous sections, we explained our findings by referencing several existing models of free recall and recognition. However, none of these models could comprehensively account for the differences we observed in both free recall and recognition tasks. To address this gap, we propose a bilingual cognitive efficiency framework that explains the dissociations between recall and recognition based on language proficiency.

The core premise of this framework is that the allocation of cognitive resources to different levels of lexical processing varies according to language proficiency. It is widely accepted that the lexical processing of written words is hierarchically organized across multiple levels, ranging from orthographic or phonological processing to conceptual processing (Bock & Levelt, 1994; Perfetti & Stafura, 2014; Jescheniak & Levelt, 1994; Caramazza, 1997; T. O. N. Dijkstra et al., 2019). Furthermore, L2 lexical processing is generally less efficient than L1 processing (Cop et al., 2015; Dirix et al., 2020; Whitford & Titone, 2012). Specifically, processing a word in the second language (L2) typically requires more time and cognitive resources at lower levels of lexical processing, such as recognizing the visual features of letters and retrieving orthographic or phonological forms (Miller & Keenan, 2011; T. Dijkstra & van Heuven, 2002). In contrast, these lower-level processes are almost automatic for words in the first language (L1), allowing more cognitive resources to be directed toward conceptual-level processing (Dirix et al., 2020; T. Dijkstra & van Heuven, 2002). This may facilitate the spread of activation to conceptually related nodes or pre-existing experiences. Consequently, word exposure during learning is likely to boost familiarity with the L2 word form to a greater extent than with L1 words. Conversely, for L1 words, exposure in the learning phase may elicit a larger “fan effect” at the conceptual level.

In addition, during memory retrieval, as discussed earlier, word form and conceptual representation play different roles in recognition and recall (Hunt & Einstein, 1981; Mandler, 1980; Durgunoǧlu & Roediger, 1987). Recognition memory relies heavily on familiarity with the word form, whereas free recall is more dependent on conceptually driven processes. As a result, the significant increase in L2 word form familiarity due to experimental exposure would disproportionately enhance L2 recognition performance. Meanwhile, the larger fan effect for L1 words—due to more widespread conceptual activation—can increase interference, leading to more false recall and false recognition for L1 words.

Our level-based bilingual cognitive resource account addresses the language proficiency paradox by considering both the specificity of bilingual language encoding and the distinct demands of different memory measures, offering new insights into this issue.

However, in the current study, a between-subject design was employed. It would be valuable to explore whether these results would differ using a within-subject design, where the same participants view a mixed list of both L1 and L2 words. Previous studies investigating language frequency effects on free recall have demonstrated a phenomenon known as the “mixed-list paradox”, where the high-frequency advantage observed in pure-list designs is diminished, absent, or even reversed in mixed-list recall tasks (Ozubko & Joordens, 2007; Popov & Reder, 2020; Popov et al., 2019; Watkins et al., 2000). Moreover, prior research has shown that language switching at encoding stage imposes processing costs on language comprehension and production, with these costs being asymmetrical for L1 and L2 words (Bobb & Wodniecka, 2013; Macizo et al., 2012; Meuter & Allport, 1999; Thomas & Allport, 2000). Future studies could investigate level-based bilingual cognitive resources to identify whether such asymmetric switching costs influence word recognition and recall performance.

Additionally, the present study focused solely on young participants aged 17 to 22, all of whom began learning English before the age of 12. The findings may differ for younger and older learners due to age-related changes in working memory capacity (Mattay et al., 2006; Caplan & Waters, 2005; Chevalère et al., 2020), processing speed (Caplan & Waters, 2005; Perbal et al., 2002; Manard et al., 2014), and L1 interference (Calabria et al., 2015; Green & Gabrys-Barker, 2018), among other factors. Therefore, future research should explore the influence of age on bilingual proficiency effects on memory. Furthermore, as the majority of participants in this study were female, it remains unclear whether gender also plays a role in L2 word memory and recognition. Investigating the potential effects of gender in future studies could provide valuable insights into this area.

### 5.5. L1 and L2 Word Recognition in Chinese ESL Learners

Our findings offer insights into the role of orthographic features in modulating language proficiency effects on word memory. Previous research on this topic has primarily focused on alphabetic languages (e.g., L1 English and L2 Spanish), where graphic symbols represent phonemes (Vander Beken & Brysbaert, 2018; Francis & Gutiérrez, 2012; Francis & Strobach, 2013). In contrast, the present study investigates L1 Chinese, a logographic language, and L2 English, an alphabetic language. In logographic systems, symbols correspond to individual morphemes (Tzeng & Hung, 1978; Wang, 1973), directly conveying meaning rather than representing phonology. Previous research has suggested that speakers of alphabetic languages (e.g., English, French, Spanish) rely more heavily on phonological information during word recognition, as the alphabet is a sound-based script (Barron, 1986; Chikamatsu, 1996; Frost, 1998, 2012; Koda, 1996). Conversely, Chinese speakers depend more on visual information in recognizing and recalling Chinese words due to the lack of systematic grapheme–phoneme correspondence rules (Chikamatsu, 1996; Braze & Gong, 2017; Feng et al., 2001; Kuo et al., 2004; Wen et al., 2018). However, our findings indicate that differences in orthographic systems do not significantly influence word memory, as we replicated the L2 advantage in word recognition previously observed in alphabetic languages (Francis & Gutiérrez, 2012; Francis & Strobach, 2013). Nevertheless, it remains uncertain whether the same results would be observed if the modality of word presentation shifted from visual to auditory. Further research is needed to explore how orthographic and phonological processing interact over time in bilingual memory and across different modalities.

Furthermore, the differing modes of L1 and L2 vocabulary acquisition in China may also contribute to the observed L2 advantages in word recognition. Unlike bilinguals in Canada or Singapore, who acquire both L1 and L2 vocabularies through their daily lives at home and in school, Chinese English learners typically acquire L2 vocabulary primarily through textbooks or dictionaries in formal school settings, whereas L1 words are learned and used in both home and school environments (Qian, 1996). Consequently, English as a Second Language (ESL) learners in China acquire and practice L1 words in highly contextualized settings—within rich, meaningful contexts such as narratives or daily conversations. As suggested by SAC model (Buchler & Reder, 2007; Diana & Reder, 2006), retrieving an L1 word would automatically activate multiple prior contextual associations, which can lead to interference in memory retrieval for L1 words. In contrast, L2 vocabulary for Chinese ESL learners is typically acquired and practiced in a more decontextualized manner, often isolated from its contextual usage (Ünaldi et al., 2013; Qian, 1996). For instance, L2 words are usually learned by providing a definition or an L1 translation through wordlists, dictionaries, or stories from Western cultures (Nation & Nation, 2001). As a result, during the retrieval of an L2 word in experimental tasks, there is likely less interference from prior contextual associations, enhancing isolated word recognition in the process. Moreover, the decontextualized nature of L2 learning may foster a heightened focus on the formal properties of words as “language items” (Nation & Nation, 2001), such as orthography, which can also result in more habitual attention being directed toward the encoding of L2 word forms, ultimately benefiting L2 word recognition.

The observed L2 advantages in word recognition are consistent with findings from literacy studies, which suggest that decontextualized practice benefits isolated word recognition (Fleisher & Jenkins, 1978; Ünaldi et al., 2013; Oxford & Scarcella, 1994). However, it is important to note that the observed L2 advantages are likely restricted to isolated word recognition tasks and may not extend to tasks requiring higher-level cognitive processes, such as critical thinking. Heath’s (1982) seminal work on children’s bedtime routines in various U.S. communities demonstrates that decontextualized preschool literacy events can support children’s initial school learning, particularly in tasks involving knowledge of the alphabet, colors, and numbers, or identifying specific portions of words. However, this decontextualized learning approach can hinder performance in open-ended tasks, such as answering “why” or “how” questions. Similarly, Oxford and Scarcella (1994) found that while decontextualized vocabulary learning may aid learners in memorizing words for tests, it can impede the flexible use of L2 vocabulary in varied, real-world contexts.

Further research is required to examine the effects of contextualized and decontextualized learning on L2 literacy in China. Since 2018, Chinese English education has undergone significant reforms in response to government initiatives, shifting from the focus of ’opening eyes to see the world’ to ’telling Chinese stories well in English’ (Li & Yuan, 2023). These reforms aim to deepen students’ understanding of Chinese history, culture, traditions, and values (Chen, 2023). As a result, many English textbooks have been revised to replace content centered on Western culture, celebrities, and lifestyles (e.g., Easter) with topics related to Chinese traditions, festivals, and values (Liao, 2022; Fu et al., 2020). This shift promotes a more contextualized approach to language learning by encouraging Chinese ESL learners to relate textbook content to their everyday lives and express their experiences in English. However, this transition also introduces a unique challenge: Chinese ESL learners now acquire and use L2 words that are specific to Chinese culture, which are rarely known or used by native English speakers (e.g., moxibustion: traditional Chinese medicine treatment method). Understanding how Chinese ESL learners and native English speakers learn and retain these culture-specific terms could provide valuable insights into the role of contextualization in literacy development. The further exploration of this issue is essential for understanding the broader implications of such reforms on L2 literacy acquisition.

## 6. Conclusions

The study revealed a dissociation between recall and recognition tasks, with L2 advantages observed in word recognition and picture endorsement tasks. However, these effects diminished in free recall, where both languages had similar levels of correct recall, but L1 showed a higher frequency of false recall. These findings lead to four key conclusions. First, recognition tasks involve different retrieval demands: word-form processing plays a crucial role in word recognition and picture endorsement, while conceptual representations are more important for free recall. Second, lower pre-experimental exposure to L2 words and greater processing demands may enhance the distinctiveness of L2 word forms, boosting their familiarity and leading to the observed L2 advantages in correct recognition. Third, greater experimental interference, stronger conceptual associations, and the more efficient spread of activation in L1 words may contribute to the increased rate of false recall in L1. Finally, these results call for a rethinking of the language proficiency effect by adopting a more interactive perspective that considers the combined influences of pre-experimental experience, encoding efficiency, language-specific lexicons, memory storage, retrieval cues, and retrieval demands.

## Figures and Tables

**Table 1:** Language background of participants in Experiments 1, 2, and 3.

	CH List				EN List			
	Chinese History		English History		Chinese History		English History	
	*M* (*SD*)	Range	*M* (*SD*)	Range	*M* (*SD*)	Range	*M* (*SD*)	Range
Experiment 1: Word Recognition								
Age at Learning Start	1.14 (0.91)	0–3	7.97 (1.97)	3–12	1.01 (1.07)	0–4	7.52 (1.95)	3–10
Language Exposure (%time)	0.84 (0.12)	0.5–1	0.11 (0.09)	0–0.4	0.79 (0.14)	0.5–1	0.21 (0.17)	0.01–0.9
Order of Language Dominance	1 (0)	1–1	2.14 (0.35)	2–3	1 (0)	1–1	2.23 (0.43)	2–3
Self-reported Speaking Proficiency	9.28 (1.07)	6–10	5.86 (1.53)	1–8	9.29 (1.61)	4–10	6.45 (1.67)	3–10
Self-reported Understanding Proficiency	9.24 (1.15)	6–10	5.34 (1.4)	1–8	9.32 (1.4)	5–10	6.13 (1.84)	3–10
Self-reported Reading Proficiency	9.21 (0.94)	7–10	7.1 (1.32)	1–8	9.16 (1.49)	5–10	6.94 (1.31)	3–10
Self-reported Writing Proficiency	8.38 (1.61)	3–10	6.38 (1.52)	3–9	8.65 (1.7)	5–10	6.23 (1.78)	3–10
Experiment 2: Picture Endorsement								
Age at Learning Start	0.88 (0.75)	0–3	7.16 (2.07)	3–12	1.21 (0.90)	0–3	7.31 (2.48)	2–11
Language Exposure (%time)	0.81 (0.11)	0.60–0.97	0.10 (0.07)	0.01–0.30	0.80 (0.13)	0.60–0.99	0.07 (0.06)	0.00–0.03
Order of Language Dominance	1 (0.00)	1–1	2.23 (0.43)	2–3	1 (0.00)	1–1	2.21 (0.41)	2–3
Self-reported Speaking Proficiency	9.27 (1.05)	7–10	5.73 (1.85)	2–10	9.14 (1.43)	5–10	5.72 (2.09)	1–9
Self-reported Understanding Proficiency	9.18 (1.11)	6–10	5.60 (1.77)	3–10	9.34 (1.17)	5–10	5.55 (1.66)	3–9
Self-reported Reading Proficiency	8.92 (1.27)	5–10	6.87 (1.75)	3–10	9.00 (1.34)	5–10	6.97 (1.92)	3–9
Self-reported Writing Proficiency	8.58 (1.46)	6–10	5.92 (1.89)	2–10	7.93 (1.87)	3–10	6.17 (1.83)	2–9
Experiment 3: Word Recall								
Age at Learning Start	1.20 (0.93)	0–3	6.68 (2.13)	3–12	0.92 (0.80)	0–3	6.69 (2.33)	2–10
Language Exposure (%time)	0.83 (0.12)	0.50–0.99	0.09 (0.07)	0.01–0.30	0.85 (0.12)	0.50–0.99	0.10 (0.09)	0.01–0.50
Order of Language Dominance	1 (0)	1–1	2.24 (0.44)	2–8	1 (0)	1–1	2.08 (0.26)	2–3
Self-reported Speaking Proficiency	9.30 (1.11)	6–10	5.77 (1.64)	2–10	9.28 (1.02)	6–10	6.34 (1.56)	1–10
Self-reported Understanding Proficiency	9.33 (1.11)	6–10	5.47 (1.87)	2–9	9.05 (1.05)	7–10	5.98 (1.22)	3–7
Self-reported Reading Proficiency	9.15 (1.09)	6–10	6.97 (1.33)	2–9	9.06 (0.97)	7–10	7.36 (1.06)	3–7
Self-reported Writing Proficiency	8.61 (1.52)	4–10	5.53 (1.44)	2–8	8.13 (1.43)	5–10	6.27 (1.14)	4–8

Participants self-rated their proficiency on a scale from 0 to 10. The score of language exposure ranged from 0% to 100%. The order of language dominance ranged from 0 to 5.

**Table 2:** Model summaries for semantic judgement task: accuracy and Rts in Exp. 1, Exp. 2, and Exp. 3.

Dependent Variable	Fixed Effect	Estimated Coefficient	Standard Error	*z*	*p*
Experiment 1					
Accuracy	Intercept	2.74	0.19	14.21	***
	Language	−1.01	0.31	−3.28	**
Rts	Intercept	1.00	0.02	54.83	***
	Language	0.23	0.03	6.71	***
Experiment 2					
Accuracy	Intercept	3.49	0.26	13.66	***
	Language	−1.19	0.37	−3.22	**
Rts	Intercept	1.01	0.02	49.19	***
	Language	0.30	0.04	7.99	***
Experiment 3					
Accuracy	Intercept	3.30	0.25	13.27	***
	Language	−0.69	0.29	−2.37	*
Rts	Intercept	0.87	0.02	45.21	***
	Language	0.25	0.03	8.74	***

*** *p* < 0.001, ** *p* < 0.01, and * *p* < 0.05.

**Table 3:** Model summaries for memory tests in Exp. 1, Exp. 2, and Exp. 3.

Dependent Variable	Fixed Effect	Estimated Coefficient	Standard Error	*z*	*p*
Exp. 1: Word Recognition					
Rts (experimental items)	Intercept	0.95	0.02	40.73	***
	Language	−0.06	0.05	−1.24	0.22
Rts (foil items)	Intercept	1.05	0.03	34.54	***
	Language	0.02	0.06	0.39	0.70
Exp. 2: Picture Endorsement					
Rts (experimental items)	Intercept	1.22	0.03	39.64	***
	Language	0.02	0.05	0.29	0.769
Rts (foil items)	Intercept	1.49	0.04	33.66	***
	Language	0.12	0.08	1.49	0.142
Exp. 3: Free Recall					
Accuracy	Intercept	−1.24	0.20	−6.36	***
	Language	0.35	0.27	1.31	0.192

*** *p* < 0.001.

**Table 4:** Mean (SE) memory performance in Experiments 1 and 2.

	Hit Rate	FA Rate	d’
Exp1: Word Recognition			
CH list	0.72 (0.02)	0.17 (0.01)	1.63 (0.07)
EN list	0.87 (0.01)	0.11 (0.01)	2.58 (0.09)
Exp2: Picture Endorsement			
CH list	0.66 (0.02)	0.25 (0.02)	1.21 (0.07)
EN list	0.79 (0.01)	0.18 (0.02)	1.87 (0.09)

## Data Availability

Data and the model comparisons performed can be found at https://osf.io/j4sbh/?view_only=17f762e7b8b7410485c11b4bb4f3f2e7, accessed on 23 February 2025.
